# Genomic resequencing reveals genetic diversity, population structure, and core collection of durian germplasm

**DOI:** 10.1038/s42003-025-08715-3

**Published:** 2025-08-23

**Authors:** Yiwang Zhong, Liying Feng, Huidong Deng, Xiaohao Ji, Jing Zhang, Yangyang Sun, Peifan Lin, Yang Qiao, Shenghua Xie, Haibo Wang, Lijun Guo, Xuejie Feng

**Affiliations:** 1 Sanya Institute, Hainan Academy of Agricultural Sciences, Sanya, China; 2 Institute of Tropical Fruit Trees, Hainan Academy of Agricultural Sciences, Haikou, 571100 China; 3 Yazhouwan National Laboratory, Sanya, China; 4 Key Laboratory of Genetic Resources Evaluation and Utilization of Tropical Fruits and Vegetables (Co-construction by Ministry and Province), Ministry of Agriculture and Rural Affairs, Haikou, China; 5 Research Institute of Pomology, Chinese Academy of Agricultural Sciences, Xingcheng, 125100 China; 6 Key Laboratory of Horticultural Crops Germplasm Resources Utilization, Ministry of Agriculture and Rural Affairs of the People’s Republic of China, Xingcheng, China; 7 Key Laboratory of Southern Subtropical Plant Diversity, Fairy Lake Botanical Garden, Shenzhen & Chinese Academy of Sciences, Shenzhen, China; 8 Key Laboratory of Tropical Fruit Tree Biology of Hainan Province, Haikou, China

**Keywords:** Plant genetics, Population genetics

## Abstract

*Durio zibethinus* Murr. is a tropical fruit crop of growing global importance, prized for its unique flavor and nutritional value. Yet only a narrow genetic base has been utilized in breeding efforts. We performed whole-genome resequencing of 114 diverse durian accessions collected in China, identifying over 39 million high-quality SNPs across the genome, revealing genome-wide patterns of diversity. Population structure analysis revealed three major genetic clusters, supported by PCA, phylogenetic analysis, and STRUCTURE modeling. Genome-wide scans identified candidate selective sweeps, and several genes potentially under selection. From the 114 accessions, we further identified a core collection of representative durian germplasms capturing the majority of the species’ genetic diversity. This subset includes both elite cultivars and genetically distinct individuals. Our study provides insights into the genetic diversity and population structure of cultivated durian. The defined core collection and genomic variation map establish a valuable resource for durian breeding and germplasm conservation. These findings will facilitate the identification of superior alleles for important traits and guide future durian improvement programs.

## Abstract (web summary)

Whole-genome resequencing of 114 durian accessions defines a genetically diverse core collection and reveals shared selection signals, providing foundational resources for breeding and conservation.

## Introduction

Durian (*Durio zibethinus*, 2*n* = 56) is an iconic tropical fruit tree native to Southeast Asia^1,2^. It is revered as the “King of Tropical Fruits” for its large, spiky fruits with richly aromatic, flavorful arils. Beyond cultural significance, durian is a major economic crop in its region of origin—the high market value of durian fruit makes it one of the most important agricultural commodities in countries like Thailand, Malaysia, and Indonesia^1^. In recent years, global demand for durian has surged, driven especially by Chinese markets. Thailand alone exported on average $3.3 billion worth of durians annually in 2021–2022, making it the country’s third most valuable agricultural export^3^. This booming popularity underscores durian’s prominent status among tropical fruit crops.

Durian is believed to have originated in Borneo and Sumatra. Its cultivation area extends from Sri Lanka, India, and Myanmar in the west, through Thailand, the Malay Peninsula, Borneo, Sumatra, and the Philippines, all the way to Papua New Guinea in the east^4–6^. Durian has a long cultivation history with numerous named varieties and landraces. Through extensive hybridization and farmer selection, hundreds of cultivars have been developed across Southeast Asia^7^. These cultivars display remarkable variation in tree growth habit, flowering time, fruit size, flesh color and texture, flavor and aroma intensity^5,6^. Notably, the notorious strong odor of durian fruit differs by cultivar and is linked to sulfur volatile compounds. Cultivars also differ in important agronomic traits such as yield and disease resistance^8–10^. Such trait diversity indicates an opportunity for genetic improvement—breeding superior durian cultivars with enhanced flavor profiles, disease resistance, and environmental adaptability remains a key goal for horticulturists.

Despite its economic and botanical significance, the genetic basis of many important durian traits is still poorly understood. One reason is that durian breeding and research have historically lagged behind those of other fruit crops. Many durian orchards rely on a few elite clones propagated vegetatively, which limits the genetic diversity utilized in cultivation^11^. The *Durio* genus itself comprises about 27 species worldwide^12^, but only *D. zibethinu*s is widely cultivated; several wild *Durio* species are threatened by habitat loss, which risks eroding potentially valuable genetic resources^13^. Conservation and study of durian germplasm diversity are therefore of paramount importance for both safeguarding biodiversity and expanding the breeding gene pool.

Recent advances in genomics have begun to shed light on durian’s genetic landscape^14,15^. The first durian reference genome (Musang King cultivar) was published in 2017, revealing a ~800 Mb genome and gene families associated with durian’s unique fruit odor. Follow-up analyses confirmed a whole-genome duplication in durian’s evolutionary history and catalogued thousands of resistance gene analogs^16^. Comparative genomics of a few durian cultivars has also been attempted; for instance, three Thai cultivars were recently resequenced to build a durian pangenome, showing genomic differences between Thai and Malaysian durians^1^. However, these studies focused on either a single elite genome or a small number of cultivars, and they do not capture the breadth of genetic variation present in the wider durian germplasm. In contrast, population-level analyses using molecular markers have been limited. A study in Hainan, China, using SSR markers on 32 durian accessions identified two genetic clusters and a narrow genetic base in those plantations^17^. Similarly, a chloroplast DNA (*rbcL*) analysis of durian from Borneo found low nucleotide diversity (~0.24%), indicating limited variation within regional germplasm^18^. These early efforts underscore a critical knowledge gap: a comprehensive genomic survey of durian’s genetic diversity across many accessions has not yet been reported.

Meanwhile, durian cultivation is expanding beyond its traditional range. Notably, since 2019, durian has been successfully introduced to Hainan Island in southern China, leading to a boom in commercial planting^18^. This presents both an opportunity and a challenge: the introduced germplasms in Hainan originate from various sources and could harbor untapped genetic variation, but their relationships and genetic makeup remain largely unknown. Understanding the genetic diversity and structure of these accessions is essential for systematic conservation, efficient utilization in breeding, and prevention of genetic erosion as the industry grows^17^. Additionally, defining a core collection—a representative subset capturing most of the genetic diversity—would greatly facilitate germplasm management and further research.

Here, we collected and sequenced 114 durian samples introduced in the Hainan region of China. Using the identified single-nucleotide polymorphisms (SNPs), we classified these sample resources into three subgroups based on population structure. Additionally, evident hybridization among these germplasm resources has led to insignificant genetic differentiation between geographic populations and relatively low genetic diversity. The discovery of a large number of variations not only provides a basis for a deeper understanding of the genetic evolution and structural characteristics of durian populations but also lays the groundwork for the conservation and breeding planning of introduced durian germplasm resources.

## Results and discussion

### SNP discovery and genome-wide variation

**Fig. 1:**
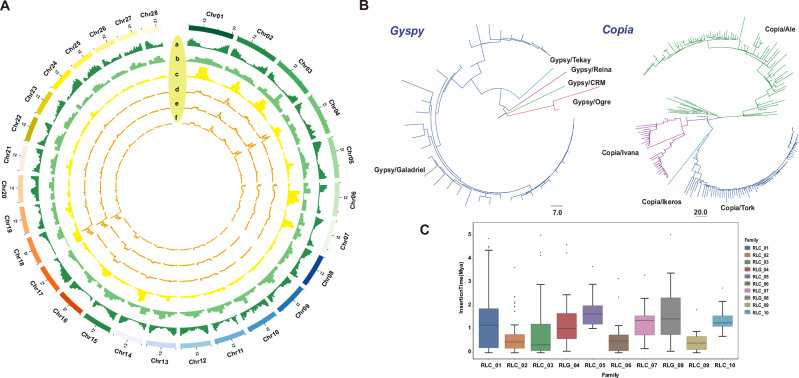
Distribution of LTR and SNPs on the genome and transposable element landscape characterization. **A** **a** Gene Density. **b** Distribution of *Copia* transposons. **c** Distribution of *Gyspy* transposons. **d** SNP Density in the POP1 population. **e** SNP Density in the POP2 population. **f** SNP Density in the POP3 population. **B** Phylogenetic tree of full-length *Gypsy* and *Copia* LTR retrotransposons based on RT domain amino acid sequences. Major clades are labeled according to known superfamilies or lineages. **C** Boxplot showing the insertion time distribution (Mya) of the top 10 most abundant LTR retrotransposon families in the durian genome. Horizontal lines within boxes indicate the median values, and dots represent outliers. RLC retrotransposon, LTR *Copia*, RLG retrotransposon, LTR *Gypsy*.

We collected a total of 114 durian samples from major cultivation areas in Hainan, China, and one site in Yunnan, southwestern China. Whole-genome resequencing of 114 durian accessions yielded a comprehensive catalog of genetic variants (Supplementary Data 1). After stringent filtering, we identified 39,266,608 high-quality SNPs distributed across the 28 durian chromosomes (Supplementary Table 1). This represents a comprehensive genomic variation dataset for durian. The SNPs covered coding and non-coding regions, providing dense markers for population genetic analysis (Supplementary Tables 2 and 3). The genome-wide variant density was not uniform: we observed higher SNP densities in certain chromosomes or regions. Notably, chromosomes 2, 4, and 18 harbored segments with elevated SNP density, whereas chromosome 10 and several late-numbered chromosomes (25–28) showed lower diversity regions (Fig. 1A). These patterns could reflect historical recombination rate variation or past selection on particular chromosomes. No large-scale gaps devoid of SNPs were present aside from centromeric or highly repetitive regions, indicating that our resequencing captured variation across most of the durian genome. Interestingly, we also observed localized gene-dense regions that appeared to coincide with putative centromeric regions. Similar patterns of gene density enrichment in centromeric areas have been reported in other plant species. For example, in the high-quality genome assembly of *Magnolia biondii*, gene-rich regions were identified in the centromeric areas of chromosomes 4, 11, and 12^19^. This suggests that such features are not unique to durian but may reflect a broader structural characteristic of certain plant genomes.

### Transposable element landscape characterization

We identified the gene content of the reference genome, as well as the abundance of *Copia* and *Gypsy* elements within LTR retrotransposons, and mapped their distribution across the chromosomes (Fig. 1A). A total of 384 intact LTR retrotransposons (LTR-RTs) were identified in the genome. Among them, the Ale and Tork lineages within the *Copia* superfamily contained more intact LTR-RTs compared to other *Copia* lineages. Similarly, within the *Gypsy* superfamily, the Galadriel lineage harbored the highest number of intact LTR-RTs. This pattern may result from the recent activity of *Copia/Ale*, *Copia/Tork*, and *Gypsy/Galadriel* members (Fig. 1B; Supplementary Fig. 1). The phylogenetic trees of the *Gypsy* and *Copia* superfamilies revealed the evolutionary relationships and activity patterns of their members. Notably, *Copia/Ale*, *Copia/Tork*, and *Gypsy/Galadriel* exhibited significant expansion (Fig. 1B). Furthermore, the insertion time analysis of intact LTR-RTs confirmed that these lineages have undergone recent lineage-specific expansions, suggesting active stress-responsive regulation and genomic rearrangements in recent evolutionary history (Fig. 1C; Supplementary Data 2).

### Population Structure and Relatedness

**Fig. 2:**
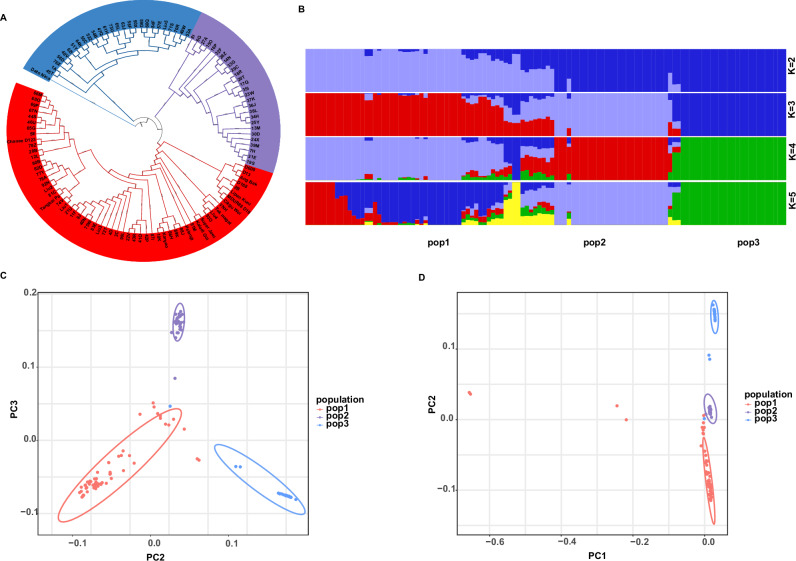
Population genetic structure and phylogenetic relationships. **A** Phylogenetic tree with bootstrap values, where red represents pop1, purple represents pop2, and blue represents pop3. **B** Population genetic structure analysis of the 114 durian accessions. **C**, **D** Principal component analysis (PCA) of all accessions.

Clustering analyses revealed that the 114 durian accessions group into three major genetic clusters, which we designate POP1, POP2, and POP3 (Fig. 2A–C). Populations POP1 and POP2 are more closely related, overlapping partially along PC2, while POP3 is more differentiated along PC1. These patterns were supported by model-based STRUCTURE analysis, which indicated an optimal *K* = 3 populations. At *K* = 3, each accession’s genome was largely assigned to one of three clusters, with only a few admixed individuals showing mixed ancestry (Fig. 2B). We mapped the origins of the accessions and found that the genetic clusters did not strictly correspond to geographic source: accessions from multiple source locations fell into the same cluster, suggesting considerable exchange of planting material among regions. This admixture is also evident in the neighbor-joining phylogenetic tree (Fig. 2A), where the three clusters are interspersed with short branch lengths between some accessions from different locales. Such results imply that hybridization and human-mediated movement of durian cultivars have blurred the simple geographic structuring of the gene pool.

To clarify the clustering and classification of the 114 durian germplasm accessions, principal component analysis (PCA) was performed based on high-quality SNPs, providing an overview of the genetic differentiation among these accessions (Fig. 2C, D). Consistent with the phylogenetic tree of the 114 accessions, the three distinct groups were clearly classified and clustered.

### Genetic diversity and linkage disequilibrium within populations

**Fig. 3:**
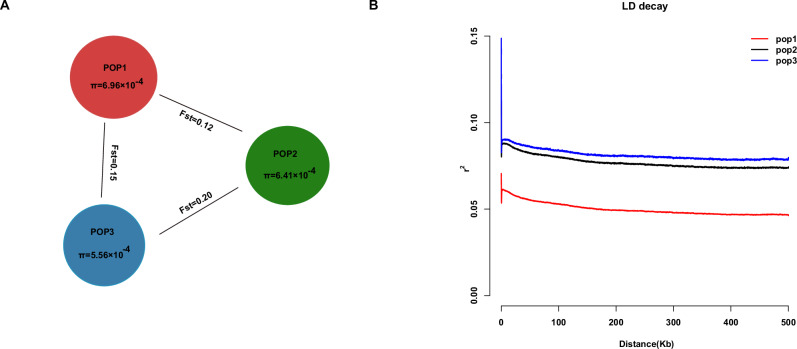
Genetic diversity and divergence of three populations. **A** The nucleotide diversity (*π*) of the three groups and the *F*_st_ values between populations. **B** LD decay of three populations. The *x*-axis represents physical distance between SNPs, and the *y*-axis represents the *r*^2^.

We compared genetic diversity metrics among the three populations (Fig. 3A; Supplementary Data 3–5). POP1 contained the largest number of accessions and exhibited the highest internal diversity (*π* ~ 0.0019), indicating it harbors a broad genetic base. Many well-known cultivars were found in POP1, suggesting it may represent a genetically rich group possibly derived from multiple origins. POP2 included 40 accessions with intermediate diversity (*π* ~ 0.0016). Interestingly, POP2 shared a substantial proportion of alleles with POP1; in fact, many SNPs common in POP1 were also present in POP2, consistent with admixture or recent divergence between these two groups. POP3 was the smallest cluster and had the lowest genetic diversity (*π* ~ 0.0012). Accessions in POP3 were more genetically uniform and formed a tight subcluster in the PCA and tree, hinting at a possible founder effect or bottleneck in their history (Fig. 2C, D).

Pairwise *F*_st_ values confirm the population relationships: POP2 vs POP3 showed the highest differentiation (*F*_st_ = 0.20), whereas POP1 vs POP2 was the least differentiated (*F*_st_ = 0.12), with POP1 vs POP3 intermediate (*F*_st_ = 0.15). These *F*_st_ values indicate moderate genetic structure—there is clear divergence between the clusters, but not extremely high (Fig. 3A; Supplementary Data 5). The moderate differentiation and the existence of admixed individuals imply that gene flow has occurred among cultivated durian lineages. Historically, farmers may have exchanged seeds or grafting material across regions, preventing the deep isolation of gene pools. This is in line with observations in other fruit trees where human cultivation reduces geographic genetic differentiation.

POP1 not only has the highest nucleotide diversity, but it also shows the fastest linkage disequilibrium (LD) decay. In POP1, the average *r*² between SNPs decays to half of its maximum within ~50 kb, and *r*² falls below 0.2 by ~200 kb, indicating a high historical recombination rate or more ancient origin of this group’s haplotypes. POP2 and POP3, in contrast, exhibited slower LD decay: in these populations, significant LD extended over longer distances (>500 kb in POP3). This suggests smaller effective population sizes or a more recent origin for POP2 and POP3. The longer-range LD in POP3 is consistent with it being a bottlenecked group—fewer recombination events in its recent past due to lower diversity (Fig. 3B). Breeders might expect that selecting within POP1 could recombine alleles more freely, whereas in POP3, many loci might be inherited as blocks due to tight linkage.

To evaluate the functional impact of genomic variation across the three populations, we classified single-nucleotide polymorphisms (SNPs) based on predicted effect categories: high, moderate, low, and modifier. The majority of variants were categorized as Modifier (Supplementary Table 4), which typically represent intergenic or intronic variants with minimal predicted impact on gene function. Variants with Low or Moderate impact—often associated with synonymous or nonsynonymous changes—accounted for a smaller proportion, while High-impact variants, which may result in disruptive effects such as stop-gain or frameshift mutations, were relatively rare. This distribution pattern suggests that most genomic variation within and among the three populations is likely to have limited functional consequences, although a subset of variants may contribute to phenotypic differences and potential adaptation.

### Signatures of selection and candidate genes

**Fig. 4:**
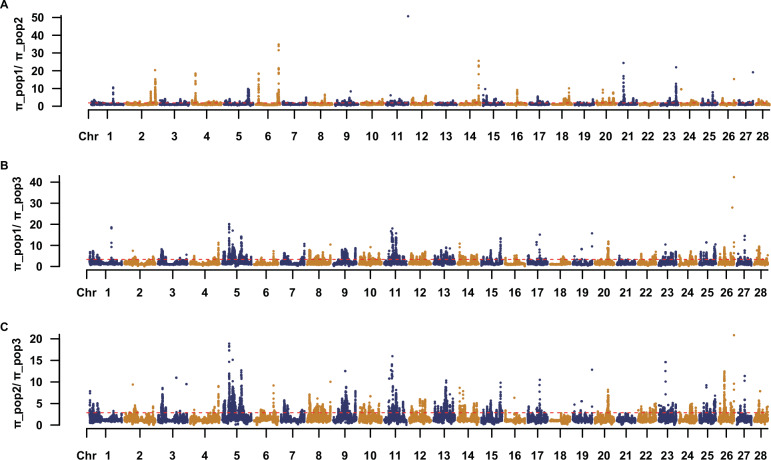
Genome-wide comparison of nucleotide diversity (*π*) ratios among three durian populations. Manhattan-style plots showing the genome-wide distribution of pairwise nucleotide diversity (*π*) ratios across 28 chromosomes for 100-kb non-overlapping sliding windows. Each dot represents the π ratio for a window, with chromosomes arranged sequentially along the *x*-axis. Dashed red lines represent genome-wide median values. Alternating point colors (orange and navy) distinguish adjacent chromosomes for clarity. **A** Distribution of nucleotide diversity ratio (π_pop1/π_pop2) across the 28 chromosomes. **B** Distribution of nucleotide diversity ratio (π_pop1/π_pop3) across the 28 chromosomes. **C** Distribution of nucleotide diversity ratio (π_pop2/π_pop3) across the 28 chromosomes.

To investigate genetic diversity differences among the three populations, we calculated and plotted the genome-wide distribution of nucleotide diversity (*π*) ratios between population pairs (Fig. 4; Supplementary Data 3). It respectively shows the *π* ratios of π_POP1/π_POP2, π_POP1/π_POP3, and π_POP2/π_POP3 across the 28 chromosomes. Overall, the *π* ratios displayed marked regional fluctuations across the genome, with distinct peaks observed in multiple chromosomal regions, suggesting localized genetic differentiation or potential selective pressures. Prominent peaks were detected on chromosomes 5, 6, 11, 21, and 26, particularly in the comparisons involving POP3 (π_POP1/π_POP3 and π_POP2/π_POP3), indicating substantial divergence between pop3 and the other two populations in these regions. Notably, some peak regions—such as those on chromosomes 6, 15, and 23 appeared in more than one population comparison, suggesting shared differentiation hotspots among populations. These elevated *π* ratio regions may represent candidate loci under selection and warrant.

Our genome scan for selection revealed multiple candidate selective sweeps in the durian genome. We focused on regions that showed extreme patterns across all analyses. One prominent example is a region on chromosome 15 where POP3 in particular showed a sharp drop in nucleotide diversity accompanied by high differentiation (Supplementary Fig. 2; Supplementary Data 4). This signals a likely selective sweep in POP3 – possibly all individuals in POP3 carry the same favored haplotype at this locus. Interestingly, POP1 and POP2 also showed reduced diversity in that region, though not as extreme, suggesting the sweep may have occurred before those groups split or due to a shared selection pressure.

### Core collection of durian germplasm

Based on our diversity and clustering results, we selected a core collection of durian germplasm that encapsulates the majority of genetic diversity found in the full set of 114 accessions. The core collection consists of 26 representative accessions (Supplementary Table 1). This subset was chosen using a diversity-maximization strategy to ensure inclusion of unique alleles and distant lineages. All three genetic populations (POP1–POP3) are represented in the core. Specifically, we included 10 accessions from POP1, 8 from POP2, and 8 from POP3, reflecting the relative diversity and size of those groups. Within POP1, which had many similar individuals, we picked those spanning the range of the cluster’s variation. In POP3, given its lower diversity, we included a slightly higher fraction of individuals to capture its alleles.

Practically, this core set can serve as a manageable collection for conservation and breeding programs. It provides a focused subset of germplasm on which more detailed evaluations can be done without having to maintain all 114 accessions at all times. Moreover, by capturing maximal allelic diversity, the core ensures that breeders or researchers can access almost the full genetic repertoire of the collection by working with this subset. For example, the core includes famous cultivars like D24 and Musang King, which are known for excellent fruit quality, as well as lesser-known genotypes that harbor unique traits. This combination of well-known germplasm in the core could be particularly powerful for breeding— crossing between divergent core members may yield hybrids that combine desirable attributes from different lines.

The construction of a durian core collection addresses a key objective of our study: balancing conservation and utilization. This core set lays the groundwork for a durian genetic improvement platform in Hainan and beyond, facilitating international exchange of germplasm and knowledge while minimizing redundancy.

### Shared genes under selection across populations

**Fig. 5:**
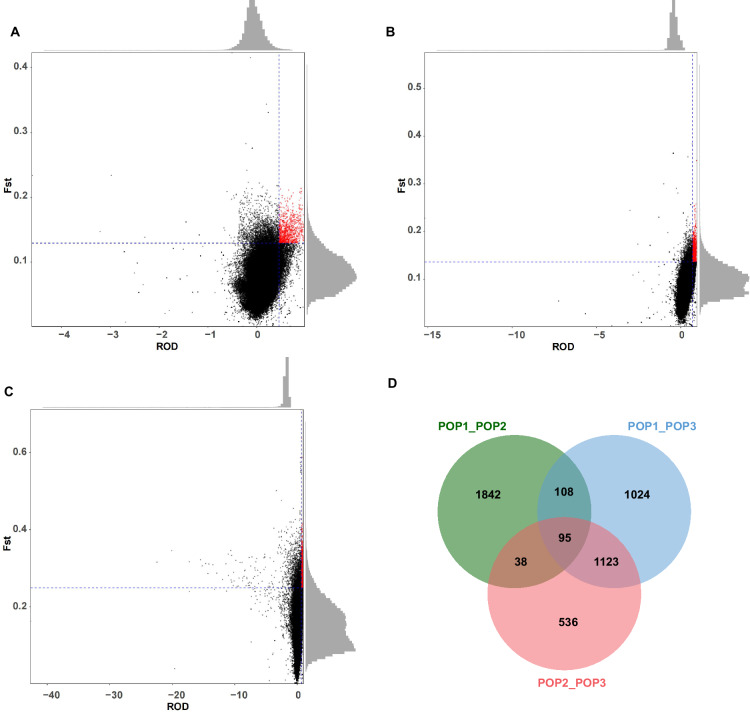
Selected genes were identified in three durian populations. **A**–**C** Joint distribution plots of Fst and ROD for pairwise comparisons among populations: **A** POP1 vs. POP2, **B** POP1 vs. POP3, and **C** POP2 vs. POP3. Each dot represents a 100-kb genomic window. Red dots indicate windows within the top 5% of both Fst and ROD values, considered candidate regions under positive selection. Marginal histograms show the distribution of *F*_st_ and ROD for each comparison. Blue dashed lines represent the 95th percentile cutoffs for each metric. **D** The number of overlapping and unique candidate genes under selection among the three population comparisons.

In total, 95 genes were identified as candidates of selection that are shared among all three populations (Fig. 5; Supplementary Data 6). Shared selective signals suggest common domestication or improvement targets—traits that durian farmers consistently favored regardless of region. Functional annotation of these genes highlighted several categories of interest. Functional annotation of genes under selection revealed several key regulatory families, including PPR, MYB, and bZIP, which are involved in stress responses, developmental processes, and transcriptional control. The presence of FAD-dependent oxidoreductases suggests a role in fatty acid metabolism and aroma-related volatile synthesis, while apoptosis regulators may contribute to programmed cell death associated with fruit ripening or stress adaptation. These findings indicate that traits related to environmental resilience, fruit quality, and metabolic regulation were recurrent targets during durian domestication and improvement.

It is worth noting that while we term these “genes under selection” further validation is needed to confirm causative links to traits. Nonetheless, the overlap of selection signals across independent populations strengthens the evidence that these loci were important in durian’s cultivation history. The breadth of functions—from flavor/aroma to disease resistance—highlights the multidimensional nature of durian domestication, where growers likely balanced fruit quality traits with survivability traits. Our list of candidate genes provides a valuable starting point for future functional studies and marker development. For example, markers in these genes can be tested for association with phenotypic variation in durian, and superior alleles could be employed in breeding or genome editing to improve varieties.

### Comparison with previous studies

Our genomic resequencing of 114 durian accessions provides a view of genetic diversity and structure in this tropical fruit tree. Prior to this work, studies of durian genetic diversity were limited to smaller sample sizes or lower-resolution markers^20,21^. A research team in Malaysia conducted a study on the genetic variation of 27 collected durian types using simple sequence repeat (SSR) markers, demonstrating that SSR markers are effective in revealing genetic differences among durian types and challenging the current morphology-based classification system^21^. A recent study in Hainan used 32 durian accessions and a set of SSR markers to infer population structure. They reported two sub-populations and noted that many accessions were essentially duplicates of each other, reflecting a narrow genetic base in the sampled plantations^17^. In contrast, our SNP-based genome-wide analysis with over 100 accessions reveals a more nuanced structure of three genetic groups and detects admixture between them. The higher resolution of millions of SNPs allows us to distinguish subgroups that SSRs might have clustered together and to quantify the degree of genetic differentiation more precisely. Interestingly, the SSR study’s finding of one cluster overlapping heavily with another is mirrored in our results: we found POP1 and POP2 to be closely related with substantial allele sharing. This suggests that despite different methodologies, both studies consistently indicate a lack of deep divergence among the cultivated durian gene pool. Our work builds on these earlier findings by expanding the sample size and genome coverage, thereby uncovering an additional group (POP3) and providing clearer evidence of hybridization and gene flow among domesticated durians (Figs. 2 and 3).

Another comparative point is the overall genetic diversity observed. The SSR study and an Indonesian chloroplast DNA study both implied low genetic diversity in durian germplasm^18^. We also found relatively modest nucleotide diversity (*π* ~ 0.002) and strong linkage disequilibrium in certain groups, consistent with a domestication bottleneck effect. However, the diversity in our panel is not uniformly low—POP1, presumably containing more ancestral or mixed lineages, retains considerable variation. It suggests that while those initially planted in Hainan local orchard populations may be genetically narrow if they originated from a few popular varieties, the global durian germplasm is richer if one accesses multiple sources. Our results advocate for germplasm exchange and introduction with proper biosecurity, as bringing in genetically distinct durian varieties can significantly broaden the breeding gene pool.

In the broader context of durian genomics, our study complements recent genomics research on this species. Pootakham assembled genomes of three Thai durian cultivars and constructed a durian pangenome, highlighting structural variation and differences between Thai and Malaysian cultivars^1^. While their focus was on deep genome characterization of a few lines, our focus is on shallow genome sampling of many lines. Together, these approaches offer a comprehensive picture: the pangenome approach uncovers presence/absence variations and lineage-specific genes, whereas our population resequencing uncovers allele frequency dynamics and population-level selection. Notably, Pootakham et al. also reported that durian genomes have expansions in certain gene families and that cultivars differ in those aspects^1^. Moreover, the evidence from both studies suggests that durian has undergone unique genomic changes, which may require breeding strategies that account for its complex genome.

It is also insightful to compare durian’s population structure with other tropical fruit trees. Crops like mango, jackfruit^22^, and rambutan^23^, which share a similar domestication syndrome such as long-lived, outcrossing trees, traditionally propagated by seed or grafting of elite clones, often show relatively weak population structure and moderate diversity in cultivated forms^24,25^. Human selection tends to be local for fruit quality, but the movement of planting material and cross-pollination blur genetic lines. Our finding of only moderate *F*_st_ (0.12–0.20) between durian populations aligns with this general pattern (Fig. 3). It contrasts with annual selfing crops or regionally isolated landraces^26–28^. Durian’s perennial nature and long generation time might have also slowed down differentiation; plus, many named durian varieties in Southeast Asia have complex pedigrees with contributions from multiple geographic sources^29^, which could explain the mosaic ancestry we observe.

### Implications for durian breeding and conservation

Our study carries several practical implications for durian improvement and germplasm management. First, the genomic variation map and SNP resource we generated is a valuable foundation for molecular breeding. The availability of ~39 million SNPs enables the development of a high-density genotyping array or targeted marker panels for durian (Supplementary Table 1). Breeders can utilize these markers for genomic selection, association mapping of traits, and routine fingerprinting of cultivars. For example, traits like fruit flavor intensity or disease resistance, which are challenging to assess in young seedlings, could be selected for using DNA markers if genomic prediction models are built^30,31^. Having such a resource places durian on par with other fruit crops where genomic breeding is increasingly adopted.

The clarified population structure informs breeding strategy. The presence of three genetic clusters suggests that crossing individuals from different clusters could harness heterosis (hybrid vigor) due to their genetic divergence. Conversely, within-cluster crosses might be more predictable in performance (less segregating variation) but may offer limited novelty. POP3 accessions, being genetically similar, might cross well with POP1– introduce new variation and vigor into otherwise inbred lines. Our analysis also flags specific accessions that are highly admixed or genetically unique; these could be prioritized as “bridges” in breeding to combine gene pools or as reservoirs of rare alleles. From a conservation perspective, the fact that much diversity lies in POP1 suggests that germplasm from that group should be conserved ex situ and in situ, as it constitutes a rich genetic reservoir for durian.

The identification of 95 candidate genes under selection provides hypotheses about key traits under human selection. This is directly relevant to breeding. Breeders and researchers can now focus on this set of genes for functional validation. Some of the candidate genes might also be useful for marker-assisted introgression—if wild relatives or unselected germplasm have superior alleles, breeders could cross them into elite cultivars while using our SNP markers to monitor the gene’s transfer. Additionally, knowledge of these selection targets can inform conservation: we might want to ensure that alternative alleles (not just the selected ones) of these genes are preserved in germplasm banks in case they are needed for future challenges.

The establishment of a durian core collection is another major contribution with practical value. The core collection approach also helps genebank management: maintaining every accession can be costly for a tree that takes space and years to fruit, so concentrating resources on a core is efficient. Our core captures >95% of allelic diversity, which is comparable to core collections developed for other crops in terms of retention of variation. This high retention suggests minimal compromise in genetic coverage, aligning with the principle that a well-designed core can significantly reduce redundancy. Importantly, the core includes elite cultivars like D24 (a leading Malaysian clone) and promising types like D163, meaning it is not just diverse but also agronomically relevant. We anticipate that this core will be a cornerstone for durian breeding in China and beyond, as it balances diversity with manageability, an idea supported by previous authors who noted that core collections enable identification of superior alleles for crop improvement.

From a conservation standpoint, our findings highlight the need to protect durian’s genetic diversity both in the wild and in cultivation. The relatively low diversity in some cultivated groups (POP3) raises concerns that heavy reliance on a few clones could make durian vulnerable to pests, diseases, or climate change. The Irish potato famine^32^ and banana Panama disease^33^ are classic examples of the perils of narrow genetic bases in crops; durian should avoid a similar path. That is to broaden the genetic base in durian breeding populations by incorporating germplasm from underutilized sources (wild relatives or farmers’ landraces from Borneo and Sumatra, the putative center of origin)^34,35^. The genomic data can assist in this by identifying which wild accessions are most genetically distinct, hence contributing novel alleles. Additionally, breeding for disease resistance should continue to be a priority, especially as durian cultivation spreads to new environments where it may encounter different pathogens.

### Limitations and future directions

While this study represents a significant advance, we acknowledge several limitations. First, our sampling, though broad for China’s durian germplasm, may not include all major durian lineages present in Southeast Asia. The 114 accessions were largely those introduced to Hainan and Yunnan; germplasm from countries like Thailand, Malaysia, and Indonesia was indirectly represented but not exhaustively. Future work should extend sampling to wild durian populations and traditional orchards in the center of origin, to capture alleles that might be missing from the current cultivated gene pool. This would also help clarify how domestication occurred—for example, did domestication happen once or multiple times independently? Our data hint at multiple introductions merging, but a more comprehensive sampling is needed to resolve durian’s domestication history. Second, our analysis focused on single-nucleotide polymorphisms, which are abundant but not the only type of genetic variation. Structural variations (SVs) such as insertions, deletions, and copy-number changes can also have major phenotypic effects. The durian pangenome study found presence/absence variation in genes related to important traits. Incorporating SV detection in our population panel could reveal additional layers of diversity and selection. However, SV calling in a diverse set of short-read sequenced samples is non-trivial and was beyond our current scope. As long-read sequencing becomes more accessible, future studies might resequence durian accessions with long reads to capture SVs and even assemble multiple genomes for a refined pangenome.

Although we identified candidate genes under selection, we did not directly measure phenotypic traits in these accessions. Therefore, we can only speculate on trait associations. A future direction is to conduct genome-wide association studies (GWAS) for traits like fruit weight, flavor compounds, tree vigour, and disease resistance by combining our genotypic data with extensive phenotyping across different environments. This would validate which genes truly affect traits of interest and could uncover additional loci not detected by our selection scans (since not all trait-related genes show classic sweep signals). Controlled crosses followed by QTL mapping could similarly be useful, although the long generation time of durian makes breeding experiments challenging.

Another limitation is related to population structure in association analyses. The presence of structure in the three clusters means that naive association tests could yield false positives if not accounted for. We acknowledge that some of our candidate “selection” signals might be due to demographic history rather than true adaptive selection. We attempted to mitigate this by focusing on regions common to all populations, but it is possible that shared demographic events (like a shared domestication bottleneck) could produce similar signals. Further validation, such as examining these regions in wild durians or testing for functional differences, will be important to confirm the role of these genes.

Lastly, environmental factors and gene–environment interactions were not explicitly addressed. Durian in Hainan grows under different climate conditions compared to its native equatorial habitat. Some selection pressures observed might be environment-specific. Conducting reciprocal transplant experiments or common gardens in multiple locations could shed light on how different genotypes perform across environments, and whether certain alleles confer advantages under specific climate stresses. As durian cultivation expands, understanding genotype by environment interaction will become increasingly relevant.

## Conclusion

In conclusion, this study provides a comprehensive genomic assessment of durian germplasm diversity, leveraging whole-genome resequencing to illuminate patterns of variation, population structure, and selection in this important tropical fruit tree. We demonstrate that cultivated durian in our sample comprises three interrelated genetic groups with overall moderate diversity and evidence of admixture. We highlight a set of candidate genes likely involved in domestication or improvement (spanning traits of fruit quality and stress tolerance), which offers valuable targets for future research and breeding. We also deliver a practical outcome in the form of a core collection, a toolkit for conservation and genetic enhancement of durian. Taken together, our findings enrich the scientific understanding of durian’s genomics and provide actionable knowledge for breeders and conservationists. By integrating diverse germplasm and modern genomic tools, we pave the way from “omics to orchard” for durian, helping to ensure that the King of Tropical Fruits can be sustainably cultivated and improved for generations to come.

Ultimately, as durian continues to gain global prominence, harnessing its genetic diversity will be key to addressing emerging challenges—whether it be breeding trees that can thrive in new environments or developing varieties with the impeccable taste that durian lovers around the world crave. The genomic resources and insights from this study form a strong foundation for those endeavors, marking a significant step forward in durian research and its journey from the rainforests of Borneo to orchards worldwide.

## Materials and methods

### Plant materials and DNA sequencing

We sampled a total of 114 durian (*D. zibethinus*) accessions from major cultivation sites in Hainan, China, and one site in Yunnan (Southwest China). These accessions represent a wide range of germplasm introduced from Southeast Asia and maintained in Hainan’s durian germplasm nurseries (Supplementary Table 1). Young, healthy leaves were collected from each accession and flash-frozen or kept at −20 °C prior to DNA extraction. Genomic DNA was extracted using a cetyltrimethylammonium bromide (CTAB) protocol^36^.

Genomic DNA (0.2 μg per sample) was sheared to ~350 bp fragments using ultrasonication and prepared into sequencing libraries using the Rapid Plus DNA Library Preparation Kit for Illumina (RK20208, ABclonal, China). Libraries were end-repaired, A-tailed, ligated with full-length Illumina adapters, and PCR-amplified. Purification was performed with the AMPure XP system (Beckman Coulter, USA). Library quality was assessed using a Qubit® 3.0 Fluorometer (Invitrogen, USA) and an Agilent 2100 Bioanalyzer (Agilent Technologies, USA), and quantified by qPCR (>2 nM). Cluster generation was conducted on the Illumina cBot system using the PE Cluster Kit, followed by 150 bp paired-end sequencing on the Illumina HiSeq X Ten platform (Illumina, USA), and each sample was sequenced to a target depth of approximately 10× coverage of the ~800 Mb durian genome.

### Read mapping and SNP calling

Raw sequencing reads (150 bp paired-end) were processed with Fastp (v0.20.0)^37^ for quality control. Low-quality reads and adapter sequences were removed, using filters of -q 5 -u 50 -n 10 to trim bases with Phred quality <5, cut off reads with >50% low-quality bases, and discard reads with >10% unknown nucleotides. Clean reads from each accession were then aligned to the durian reference genome (‘Kanyao’ cultivar assembly)^15^ using BWA-MEM (v0.7.17)^38^ with default parameters. The resulting SAM alignments were converted to BAM format, sorted, and PCR duplicates were marked using Samtools (v1.9)^39^. We indexed the cleaned BAM files and performed variant calling with GATK HaplotypeCaller (v4.2.0.0)^40^ in GVCF mode for each sample. All per-sample GVCFs were then combined using GATK’s GenotypeGVCFs to jointly genotype across the 114 accessions, producing a multisample VCF. We applied standard hard-filters to obtain high-confidence SNPs: variants with low genotype quality (GQ) or read depth, high strand bias, or other sequencing artifacts were removed using recommended GATK filters. We further filtered out SNPs with >20% missing data across samples or minor allele frequency (MAF) below 0.01 to focus on informative polymorphisms. After filtering, a total of 39,266,608 high-quality SNPs, representing a dense genome-wide set of variants^41^. PLINK (v1.9)^42^ was used to perform additional filtering and variant selection, generating a refined VCF file for downstream analysis.

### LTR identification

Transposable elements (TEs) were annotated using the EDTA pipeline (v2.0)^43^ with the parameter --species others to enable de novo identification across all TE classes. The workflow integrates structural and homology-based tools, including LTRharvest^44^, LTR_FINDER^45^, LTR_retriever^46^, RepeatModeler^47^, and RepeatMasker^48^, allowing comprehensive detection and classification of intact and fragmented elements. The analysis was performed with the command: EDTA.pl --genome genome.fa --species others --sensitive 0 --anno 1 --threads 8. High-confidence TEs were classified into major superfamilies, and nested or low-quality predictions were filtered out. Final annotations were exported in GFF3 format for downstream analysis. The chromosomal distribution of LTR elements and SNPs across populations was visualized using Circos^49^. Reverse transcriptase (RT) domain protein sequences from *Copia* and *Gypsy* LTR retrotransposons were extracted based on the EDTA (v2.0)^43^ annotation and manually curated for completeness. Multiple sequence alignment was performed using MAFFT (v7.490)^50^. The aligned sequences were used to construct a phylogenetic tree using the neighbor-joining (NJ) method in MEGA11^51^.

### Genetic diversity and population structure analysis

Filtered SNPs were subjected to linkage disequilibrium (LD)-based filtering for population structure analysis. The selected SNP-containing VCF file was converted to PHY format using the vcf2phylip.py script. Phylogenetic tree construction was performed using PHYLIP (v3.697)^52^, with the parameters set as follows: seqboot.bar (R 100 Y 9), dnadist.par (T 2 M D 100 2 Y), neighbor.par (M 100 9 Y), and consense.par (Y). The resulting phylogenetic tree was visually refined using Evolview^53^. For principal component analysis (PCA), the VCF file was converted to PLINK format using PLINK (v1.9)^42^, with the parameter --allow-extra-chr. Population structure analysis was conducted using Admixture^54^. Since the number of genetic clusters was unknown, the range of *K* values was set from 2 to *n*, allowing the software to infer population subdivisions based on the specified *K* values. Admixture calculated the maximum likelihood estimate for each *K* value, with higher likelihood values indicating a better fit to the actual population structure. In this study, *K* values were set from 2 to 5, and STRUCTURE calculations were performed for each *K* value to infer the genetic structure of the population. We calculated within-population genetic diversity (*π*) and between-population divergence. Pairwise fixation indices (Fst) were computed using VCFtools (v0.1.16)^55^ to gauge the level of genetic differentiation between each pair of populations.

### Linkage disequilibrium and demographic inference

We evaluated linkage disequilibrium (LD) decay in each durian population by calculating the correlation coefficient (*r*²) between pairs of SNPs at varying distances. For each population, *r*² was averaged in bins of physical distance, and the decay of LD with distance was plotted. The point at which *r*² drops to half of its maximum value was used as an indicator of the LD decay rate, reflecting historical recombination and effective population size. Faster LD decay implies higher recombination or more diverse haplotypes in that population. We also used population allele frequency spectra and the extent of diversity to infer whether any populations showed signs of past bottlenecks or expansions.

### Detection of selective sweeps and candidate genes

To identify genomic regions and genes under selection, we scanned the genome for signatures of selective sweeps. Two complementary approaches were used: (1) Population differentiation (*F*_st_) outlier analysis: We looked for genomic windows 50 kb windows sliding across the genome) with exceptionally high *F*_st_ values between populations, indicating loci that differ markedly and might have been targets of divergent selection. (2) Reduction in genetic diversity: We calculated the ratio of nucleotide diversity (*π*) between populations (or compared *π* in each population to the overall *π*) to find regions of unusually low diversity in one or more populations, a hallmark of recent selective sweeps. We also computed Tajima’s *D* in sliding windows to detect deviations from neutral allele frequency spectra. Regions simultaneously showing high between-population Fst, low within-population diversity, and extreme Tajima’s *D* were flagged as candidate sweep regions. Functional annotation of genes was performed using eggNOG-mapper v2^56^ based on the eggNOG orthology database.

### Statistics and reproducibility

No statistical tests were used in this study, as the analyses were based on whole-genome resequencing and population-level genomic comparisons. Genotype data were generated from 114 biologically independent durian accessions. Each accession represents a distinct individual sampled from geographically diverse cultivation areas. Variant calling, population structure, and diversity analyses were performed using standard bioinformatics tools with default parameters as described. All analyses were conducted using publicly available software, and the results were consistent across the dataset. No experiments were repeated, as the data were not generated from replicated experimental conditions but rather represent natural genetic variation across independent samples. Where applicable, population genetic statistics such as nucleotide diversity (*π*), fixation index (*F*_st_), and linkage disequilibrium decay (*r*²) were calculated using VCFtools (v0.1.16)^55^ and custom scripts. Parameters and sample groupings are provided in the “Methods” and Supplementary Tables. No formal hypothesis testing was conducted.

## Supplementary information

Supplementary Information

Description of Additional Supplementary Files

Supplementary Data 1-6

nr-reporting-summary

## Data Availability

The raw resequencing data for 114 durian germplasm resources have been deposited in the Genome Sequence Archive^57^ (GSA; Genomics, Proteomics & Bioinformatics, 2017) at the BIG Data Center, Beijing Institute of Genomics, Chinese Academy of Sciences, under the accession number PRJCA041512. The reference genome sequence files are available at Figshare under the link (https://figshare.com/articles/dataset/Durian_genome_annotation/25237591). Genotype data in Variant Call Format (VCF) are publicly available in the Figshare database under accession code 29665418 (CC BY 4.0). The source data for Fig. 1C can be found in Supplementary Data 2. All other relevant data are available from the corresponding author upon reasonable request.
