# Supplementary material for: Genomic resequencing reveals genetic diversity, population structure, and core collection of durian germplasm
Source: Commun Biol. 2025 Aug 23;8:1273. doi: 10.1038/s42003-025-08715-3 (PMC12375001; doi:10.1038/s42003-025-08715-3)
Supplement: Supplementary file 1 — Supplementary Information [file 42003_2025_8715_MOESM1_ESM.docx]

**Supplementary Information**

Supplementary Information contains 2 figures and 4 tables.

**Supplementary Figures**

**Supplementary Figure 1**. Characterization of LTR retrotransposons in the genome.

**Supplementary Figure 2**. Genome-wide distribution of Tajima’s D values across three populations. Each data point represents a non-overlapping window across the genome. Chromosome numbers are shown on the x-axis, and Tajima’s D values are shown on the y-axis.

**Supplementary Tables**

**Supplementary Table 1**.Summary of SNPs and Indels in three populations based on genome.

**Supplementary Table 2.** Number of effects by type in all population.

**Supplementary Table 3.** Number of effects by region in all population.

**Supplementary Table 4.** Effects of genome variability in three populations.

**Supplementary Figures**


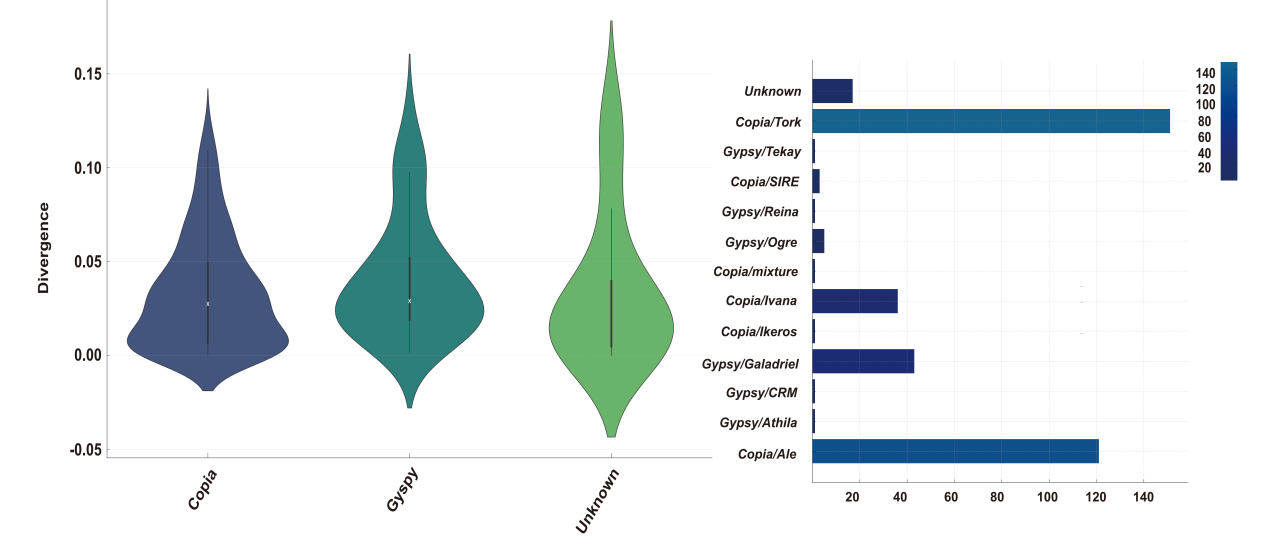


**Supplementary Figure 1**. Characterization of LTR retrotransposons in the genome.


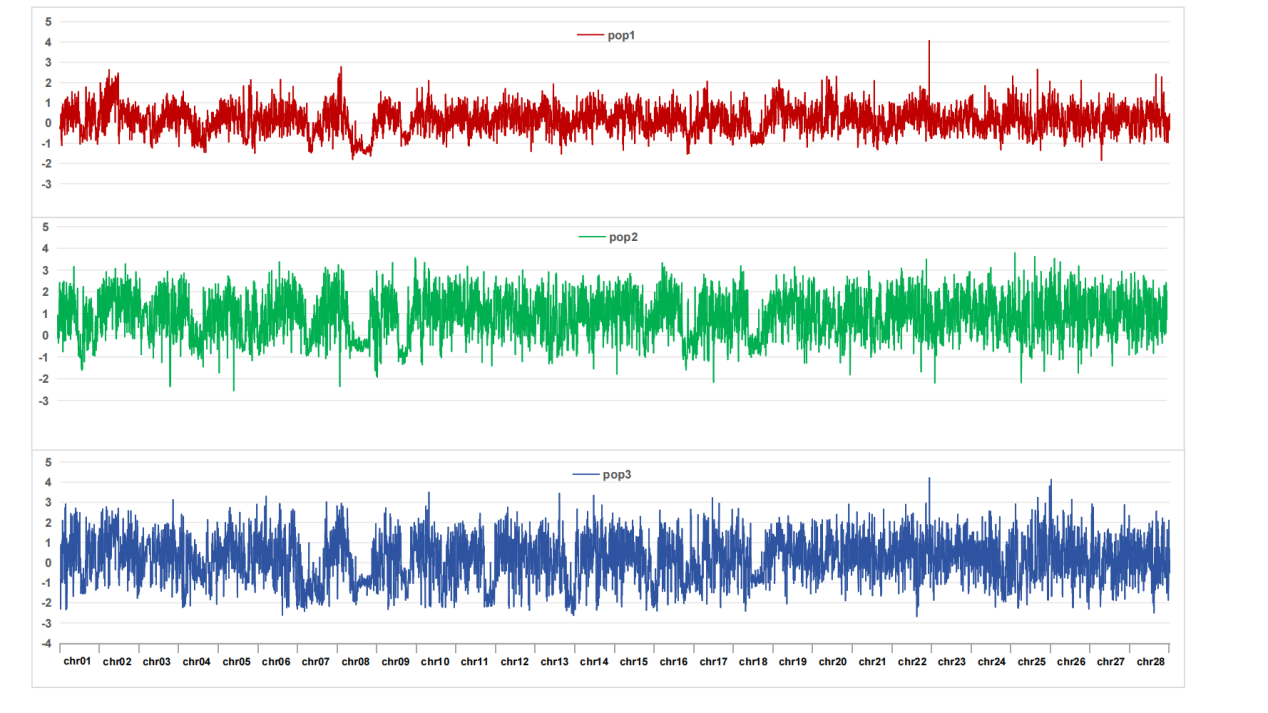


**Supplementary Figure 2**. Genome-wide distribution of Tajima’s D values across three populations. Each data point represents a non-overlapping window across the genome. Chromosome numbers are shown on the x-axis, and Tajima’s D values are shown on the y-axis.

**Supplementary Tables**

**Supplementary Table 1.** Summary of SNPs and Indels in three populations based on genome.

|  | **All variations** | | | | **High variations** | | | |
| --- | --- | --- | --- | --- | --- | --- | --- | --- |
|  | **Total** | **SNPs** | **Indels** | **Ts/Tv** | **Total** | **SNPs** | **Indels** | **Ts/Tv** |
| All Pop | 65,406,604 | 60,433,736 | 4,972,868 | 3.0281 | 41,548,219 | 39,266,608 | 2,281,611 | 3.0527 |
| Pop1 | 60,338,567 | 55,929,203 | 4,409,364 | 2.9982 | 36,117,155 | 34,203,195 | 1,913,960 | 3.0331 |
| Pop2 | 22,982,514 | 21,548,683 | 1,433,831 | 2.8439 | 15,849,219 | 14,993,974 | 855,245 | 2.9179 |
| Pop3 | 22,995,479 | 21,493,654 | 1,501,825 | 2.7748 | 12,767,958 | 12,074,737 | 693,221 | 2.8716 |

**Supplementary Table 2.** Number of effects by type in all population.

| **Type (alphabetical order)** | **Count** | **Percent** |
| --- | --- | --- |
| 3_prime_UTR_truncation | 8 | 0% |
| 3_prime_UTR_variant | 379,050 | 0.38% |
| 5_prime_UTR_premature_start_codon_gain_variant | 28,857 | 0.03% |
| 5_prime_UTR_truncation | 22 | 0% |
| 5_prime_UTR_variant | 270,867 | 0.27% |
| bidirectional_gene_fusion | 2 | 0% |
| conservative_inframe_deletion | 9,790 | 0.01% |
| conservative_inframe_insertion | 15,629 | 0.02% |
| disruptive_inframe_deletion | 17,209 | 0.02% |
| disruptive_inframe_insertion | 16,188 | 0.02% |
| downstream_gene_variant | 14,440,669 | 14.34% |
| exon_loss_variant | 302 | 0% |
| frameshift_variant | 146,756 | 0.15% |
| gene_fusion | 4 | 0% |
| initiator_codon_variant | 297 | 0% |
| intergenic_region | 61,367,557 | 60.94% |
| intragenic_variant | 7 | 0% |
| intron_variant | 6,321,943 | 6.28% |
| missense_variant | 975,945 | 0.97% |
| non_coding_transcript_variant | 1,301 | 0.00% |
| splice_acceptor_variant | 10,659 | 0.01% |
| splice_donor_variant | 10,790 | 0.01% |
| splice_region_variant | 154,058 | 0.15% |
| start_lost | 3,932 | 0.00% |
| stop_gained | 35,511 | 0.04% |
| stop_lost | 3,755 | 0.00% |
| stop_retained_variant | 1,172 | 0.00% |
| synonymous_variant | 619,704 | 0.62% |
| upstream_gene_variant | 15,877,872 | 15.77% |

**Supplementary Table 3.** Number of effects by region in all population.

| **Type (alphabetical order)** | **Count** | **Percent** |
| --- | --- | --- |
| DOWNSTREAM | 14,440,583 | 14.37% |
| EXON | 1,820,518 | 1.81% |
| GENE | 6 | 0% |
| INTERGENIC | 61,367,557 | 61.05% |
| INTRON | 6,196,542 | 6.16% |
| SPLICE_SITE_ACCEPTOR | 8,806 | 0.01% |
| SPLICE_SITE_DONOR | 8,864 | 0.01% |
| SPLICE_SITE_REGION | 121,612 | 0.12% |
| TRANSCRIPT | 1,308 | 0.00% |
| UPSTREAM | 15,877,872 | 15.80% |
| UTR_3_PRIME | 378,975 | 0.38% |
| UTR_5_PRIME | 299,652 | 0.30% |

**Supplementary Table 4.** Effects of genome variability in three populations.

|  | **All variations** | | | | **High variations** | | | |
| --- | --- | --- | --- | --- | --- | --- | --- | --- |
|  | **High** | **Low** | **Moderate** | **Modifier** | **High** | **Low** | **Moderate** | **Modifier** |
| all Pop | 199,455 | 759,677 | 1,029,543 | 98,533,620 | 58,374 | 483,672 | 615,425 | 60,023,836 |
| Pop1 | 145,064 | 729,142 | 961,992 | 91,365,008 | 51,746 | 454,376 | 570,458 | 52,844,540 |
| Pop2 | 65,087 | 261,095 | 347,821 | 33,319,312 | 25,265 | 198,844 | 250,284 | 22,638,693 |
| Pop3 | 63,682 | 260,551 | 343,434 | 33,978,646 | 20,261 | 162,925 | 200,259 | 18,311,154 |

**Supplementary References**

1. Ou, S. et al. Benchmarking transposable element annotation methods for creation of a streamlined, comprehensive pipeline. *Genome Biol.* **20,** 275 (2019).
2. Chang, C. C. et al. Second-generation PLINK: Rising to the challenge of larger and richer datasets. *Gigascience* **4,** 7 (2015).
3. Danecek, P. et al. The variant call format and VCFtools. *Bioinformatics* **27,** 2156-2158 (2011).
4. Chen, T. et al. The genome sequence archive family: Toward explosive data growth and diverse data types. *Genomics, Proteomics & Bioinformatics* **19,** 578-583 (2021).
