# Supplementary material for: Genomic resequencing reveals genetic diversity, population structure, and core collection of durian germplasm
Source: Commun Biol. 2025 Aug 23;8:1273. doi: 10.1038/s42003-025-08715-3 (PMC12375001; doi:10.1038/s42003-025-08715-3)
Supplement: Supplementary file 2 — Description of Additional Supplementary Files [file 42003_2025_8715_MOESM2_ESM.docx]

**Description of Additional Supplementary Files**

File name: Supplementary Data 1

Description: Mapping statistics of 114 durian accessions.

File name: Supplementary Data 2.

Description: Summary of representative transposable element clusters.

File name: Supplementary Data 3

Description: Sliding window summary of SNP numbers and Pi values across in three durian populations.

File name: Supplementary Data 4

Description: Sliding window summary of SNP numbers and Tajima’s D values across in three durian populations.

File name: Supplementary Data 5.

Description: Sliding window summary of Fst and ROD value across in three durian populations.

File name: Supplementary Data 6.

Description: Genomic locations of 95 candidate genes.
